# Supplementary material for: A value chain analysis of interventions to control production diseases in the intensive pig production sector
Source: PLoS One. 2020 Apr 8;15(4):e0231338. doi: 10.1371/journal.pone.0231338 (PMC7141678; doi:10.1371/journal.pone.0231338)
Supplement: S1 Appendix — (DOCX) [file pone.0231338.s001.docx]

**Appendix 1. Analytical example using economic welfare analysis**

The logic of market-level adjustments for when an intervention is adopted as it reduces production costs per unit of output leading to economic gains without affecting demand for a product at a given price and for an intervention being adopted because it increases demand for the product, is shown analytically in Fig A1, where *S*_0_ represents the supply curve before adopting an intervention. A supply ‘curve’, which here is portrayed as linear for illustration’s sake, can be regarded to represent production costs per unit of product, and the area under the supply curve is needed to cover production costs at each quantity that is being produced. Farms, and other firms, are able to produce quantity *Q_1_* at a cost which is equal to *P_1_* per unit (kg of meat), quantity *Q_0_* at a cost which is equal to *P_2_* and quantity *Q_2_* at a cost which is equal to *P_0_*. If an intervention is able to reduce production costs per unit of output, then the supply curve is shifted. Thus, when an intervention is in place, a lower price for the product is required to make a break-even profit, where the costs of production equal market revenues for the product, when compared with the situation before adopting the intervention. Hence, *S_1_* represents the supply curve after adopting an intervention which reduces production costs.


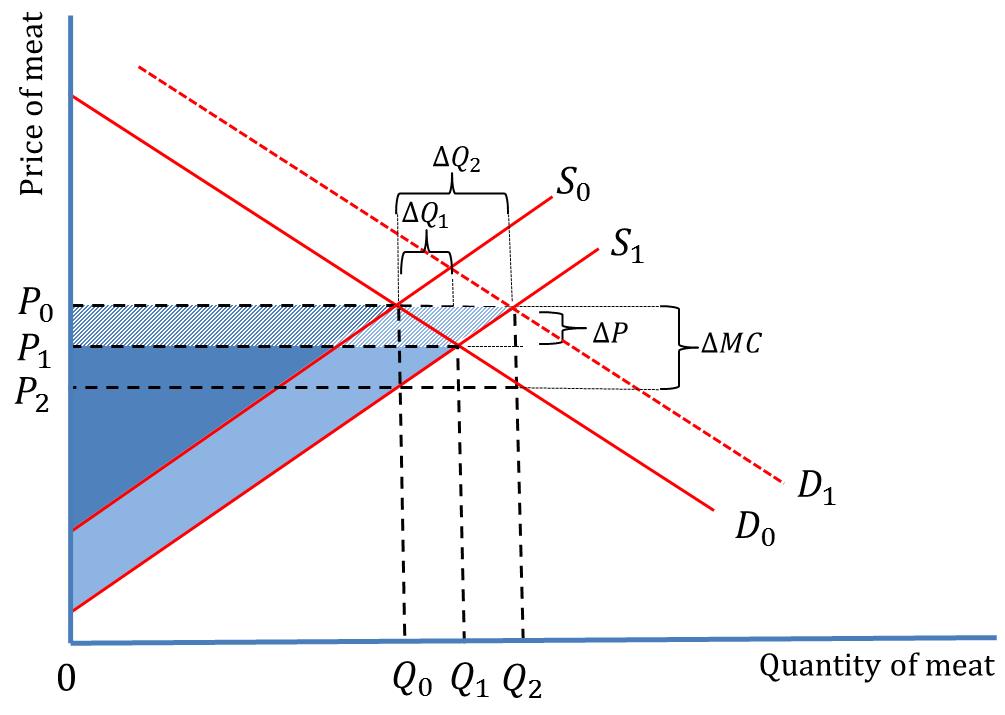


**Fig. A1. The logic of how an intervention towards reducing production diseases may influence demand for, and supply of, meat product at the aggregate level.**

*D*_0_ represents the demand curve before adopting, and after adopting, an intervention which does not result in consumers being WTP extra for quality attributes related to the intervention. Hence, the lower the price, the more consumers are willing to buy the product. For instance, at price *P_10_* consumers are willing to buy quantity *Q_1_*, while at price *P_0_* they are willing to buy only quantity *Q_0_*. Even if consumers are not WTP an extra premium for products produced using such an intervention, the quantity and price traded on the market can change along the demand curve, although this requires that the intervention changes production costs per kg of meat produced. By contrast, if consumers are WTP a premium for products produced following the use of an intervention, the demand curve can shift, for instance, from *D_0_* to location *D_1_*. In this case, quantity *Q_0_* can be traded at a higher price than *P_0_*. In fact, if production costs remain unaffected, both the price and the quantity traded can increase. Hence, *D_1_* represents the demand curve after adopting an intervention which entails that consumers are WTP a premium for the product produced with an intervention in place.

If the intervention reduces production costs per kg of meat produced, this change can be described by the change in the marginal cost of production, $\Delta MC$. This is the change associated with the adoption of the intervention. If the demand curve is unaffected, the markets will move from the previous market-clearing balance (*P_0_*, *Q_0_*) to a new balance (*P_1_*, *Q_1_*). Hence, production costs per unit of output will decrease by $\Delta MC$, but quantity traded will increase by ${\Delta Q}_{1}$ and the price of meat will decrease by $\Delta P$.

At the aggregate level, the economic welfare of consumers and producers can be measured by consumer surplus and producer surplus. In Fig. A1, consumer surplus prior to adopting an intervention is represented by the area of the triangle on the right side of the vertical line on the origin (O), below demand curve *D_0_* and above the market-clearing price *P_0_* (i.e. there are some consumers who would be WTP a higher price than *P_0_* for the product and their benefit is represented by the triangle). Producer surplus, by contrast, is represented by the area of the triangle which on the left side of the origin, above the supply curve *S_0_* and below the market-clearing price *P_0_* (i.e. there are some producers who would be willing to sell a lower price than *P_0_*, and their benefit is represented by the triangle). When assessing sector-level economic welfare effects of an intervention, we will focus on how these fields are changed as a consequence of the intervention. Hence, when an intervention produces efficiency gains so that the market-clearing balance is shifted from (*P_0_*, *Q_0_*) to (*P_1_*, *Q_1_*), consumers can gain an additional surplus which now lies below the demand curve. In Figure A1, this area corresponds to:

.

$\Delta CS=Q_{0}\Delta P+½(Q_{1}-Q_{0})\Delta P$.

Producer surplus, by contrast, changes by the amount:

.

$\Delta PS=\left( \Delta MC-\Delta P \right)Q_{0}+½(\Delta MC-\Delta P)(Q_{1}-Q_{0})$.

Change in the producer surplus consists of lost sales revenue ($Q_{0}\Delta P$) plus saved production costs ($Q_{0}\Delta MC$) and additional margin obtained ($\frac{1}{2}(\Delta MC-\Delta P)(Q_{1}-Q_{0})$ in Fig. A1). The net welfare effect to society can be obtained by taking together changes in consumer and producer surplus.

In the event that consumers are WTP an additional premium for the products produced from using the intervention, the demand curve will also shift, and the new market-clearing situation could shift. In the hypothetical example provided in Fig. A1, this is from (*P_0_*, *Q_0_*) to (*P_0_*, *Q_2_*). The market price for the product can be lower, higher or the same, before, and after, the intervention is applied. When compared to (*P_1_*, *Q_1_*), this example illustrates that, because of a premium, quantity traded can increase even if the price increases, because the old and the new product are virtually different products as they have different quality characteristics.

In Fig. A1, we have aggregated all production-side stakeholders into one group. When primary producers and food processors are separated in Fig. A1, changes in one stakeholder-group’s production costs will influence the level and shape of the overall supply curve. Depending on the shape of the primary producers’, and food processors’, supply curves, a decrease in the primary producers’ production costs can shift the supply curve of food processors. It can also influence the slope of the food processors’ supply curve.
