# Supplementary material for: A value chain analysis of interventions to control production diseases in the intensive pig production sector
Source: PLoS One. 2020 Apr 8;15(4):e0231338. doi: 10.1371/journal.pone.0231338 (PMC7141678; doi:10.1371/journal.pone.0231338)
Supplement: S2 Appendix — (DOCX) [file pone.0231338.s002.docx]

**Appendix 2. Baseline model farm scenario used in the analysis for pigmeat, 2016/17.**

|  | | **Finland** | **UK**^a^ |
| --- | --- | --- | --- |
| Number of fattening pigs | | 680 | 1 000 |
| Slaughter weight (kg, cold carcass) | | 90.6 | 81.0 |
| Meat price, €/kg | | 1.48 | 1.86 |
| Average feed price, €/t | | 211 | 289 |
| Overall Feed Conversion ratio | | 2.94 | 2.93 |
| Meat output, kg | | 211 398 | 285 114 |
| Revenue from selling meat, €/farm/year | | 312 869 | 530 313 |
| Feed costs, €/farm/year |  | 173 346 | 310 775 |
| Other variable costs, €/farm/year | | 61 305 | 82 683 |
| Labour, €/farm/year |  | 38 052 | 51 321 |
| Building and capital costs, €/farm/year | | 61 305 | 65 576 |
| Total costs, €/farm/year |  | 331 895 | 510 355 |
| Gross margin, €/farm/year | | 40 166 | 85 534 |

^a^ €1=£0.7258

Sources: [36,37,38].
