# Supplementary material for: A value chain analysis of interventions to control production diseases in the intensive pig production sector
Source: PLoS One. 2020 Apr 8;15(4):e0231338. doi: 10.1371/journal.pone.0231338 (PMC7141678; doi:10.1371/journal.pone.0231338)
Supplement: S3 Appendix — (DOCX) [file pone.0231338.s003.docx]

**Appendix 3. Summary of production and consumption side perspectives related to the characteristics of the two sets of interventions to control production diseases in pig production.**

| **Intervention** | **1) Improved hygiene in pig fattening** | **2) Enhanced care and handling of sows and piglets** |
| --- | --- | --- |
| Production perspective | Increased profit margin, growth rate and efficiency of production | Increased profit margin, except in case of feed intervention |
|  | Reduced risk of disease; disease lesions at slaughter and losses | Reduced piglet mortality; reduced need to use antimicrobials |
|  | Potential to produce more batches per year and to increase competitiveness | Improved animal welfare; better job satisfaction of farmers and farm staff |
|  | Additional labour costs associated with implementation | Possible price premium from the markets |
|  |  | More expensive feed in case of nutritional intervention; requires more labour time |
| Consumer perspective | Management intervention, not medication-based | Addresses animal psychology |
|  | Preventative, proactive measure | Positive welfare |
|  | Enhanced biosecurity can contribute to trust towards livestock production; it can also promote food safety | Proactive and relatively natural intervention |
|  | Good hygiene can also tackle concerns related to food safety | People could perceive this as very humane intervention |
|  | When adopted at large, can lead to more affordable products; can increase consumer surplus | Genetic improvements may be mixed up with GMO; hence need clear communication to the public |
| Policy issues and quality assurance | Some farms do not adopt, for reasons they have arguments for; enforcement among these farms? | Consumers appreciate feed-related interventions in pigs, hence, quality assurance schemes could have a recommendation to take into account this aspect |
|  | Could be a mandatory requirement in a quality assurance scheme | Need to have adequate staff per pig. However, unclear whether it is positive handling or human-animal interaction which leads to better results |
|  |  | Positive handling could be incorporated into welfare labelling system or standards |
| Other remarks | Perceived risk and perceived benefit are good predictors for perceived attitudes | Human-animal interaction, pro-welfare intervention; very high perceived benefits to consumers |
